# Increased Serum C-Reactive Protein Level Is Associated with Increased Storage Lower Urinary Tract Symptoms in Men with Benign Prostatic Hyperplasia

**DOI:** 10.1371/journal.pone.0085588

**Published:** 2014-01-15

**Authors:** Shun-Fa Hung, Shiu-Dong Chung, Hann-Chorng Kuo

**Affiliations:** 1 Division of Urology, Department of Surgery, Far Eastern Memorial Hospital, New Taipei City, Taiwan; 2 Department of Urology, Buddhist Tzu Chi General Hospital and School of Medicine, Tzu Chi University, Hualien, Taiwan; Eberhard-Karls University, Germany

## Abstract

**Objective:**

Chronic inflammation is considered as one of the contributing mechanisms of lower urinary tract symptoms (LUTS). Serum C-reactive protein (CRP) level is the widely used biomarker of inflammatory status. This study investigated the association between serum CRP level in men with benign prostatic hyperplasia (BPH) and lower urinary tract symptoms (LUTS) before and after medical treatment.

**Methods:**

A total of 853 men with BPH and LUTS were enrolled. All patients completed the International Prostate Symptoms Score (IPSS) questionnaire and urological examinations. The parameters of uroflowmetry (maximum flow rate, Qmax; voided volume, VV), post-void residual (PVR), total prostate volume (TPV) and transition zone index (TZI), serum prostate specific antigen (PSA), and serum CRP levels were obtained. All patients were treated with alpha-blocker or antimuscarinic agent based on the IPSS voiding to storage subscore ratio (IPSS-V/S). Correlation analyses were performed between serum CRP levels with age, IPSS, TPV, TZI, Qmax, PVR, VV, PSA and between baseline and post treatment.

**Results:**

The mean age was 66.9±11.6 years old and the mean serum CRP levels were 0.31±0.43 mg/dL. Univariate analyses revealed serum CRP levels were significantly associated with age (p<0.001), PSA levels (p = 0.005) and VV (p = 0.017), but not significantly associated with TPV (p = 0.854) or PVR (p = 0.068). CRP levels were positively associated with urgency (p<0.001) and nocturia (p<0.001) subscore of IPSS, total IPSS (p = 0.008) and storage IPSS (p<0.001) and negatively associated with IPSS- V/S ratio (p = 0.014). Multivariate analyses revealed that serum CRP levels were significantly associated with age (p = 0.004) and storage IPSS subscore p<0.001). Patients with IPSS-V/S<1 and treated with tolterodine for 3 months had significant decrease of CRP levels after treatment.

**Conclusion:**

Serum CRP levels are associated with storage LUTS and sensory bladder disorders, suggesting chronic inflammation might play a role in the patients with storage predominant LUTS.

## Introduction

International Continence Society (ICS) defined lower urinary tract symptoms (LUTS) as three categories including storage, voiding, and post-micturition symptoms [1]. In a cohort study consisting of 19,165 individuals in five countries by using the ICS definition in 2002, 62.5% of men 40 years or older reported at least one LUTS. Storage symptoms (51%) were more common than voiding symptoms (26%), and the incidence of all LUTS increased with age [2]. The causes of LUTS varied with different gender and age groups. In the male population, benign prostatic hyperplasia (BPH) was considered the dominant cause of LUTS in the population aged greater than 60 years.

Chronic inflammatory condition is a non-specific term which describes long-lasting or frequent recurrence of inflammation associated with a more specific disease. Subclinical inflammation has been reported to involve in the pathophysiology of erectile dysfunction [3], [4]. Endothelial dysfunction is believed associated with elevated proinflammatory cytokines in obese men [4], [5]. High-sensitivity C-reactive protein (hs-CRP) level is helpful in predicting the risk of cardiovascular disease and diabetes in asymptomatic people [6] as well as in men with erectile dysfunction [7].

From the histological study of the patients enrolled in the Reduction by Dutasteride of Prostate Cancer Event study (REDUCE study), chronic inflammation of the prostate was found in 77.6% of men in prostatic biopsy specimens at baseline and there is evidence of a relationship between the severity of LUTS and the degree of chronic inflammation [8], [9]. However, not all men with LUTS/BPH have urodynamically confirmed bladder outlet obstruction (BOO). Overactive bladder (OAB) symptoms in male LUTS are often caused by bladder dysfunctions alone or in combination with BOO [10].

The serum CRP level is considered as the surrogate of the chronic inflammation. In clinical practice, serum CRP levels could predict renal involvement in 44 to 83% of children with febrile urinary tract infection [11] and was significantly associated with metastatic disease in prostate cancer [12]. The associations of serum CRP level and clinical LUTS suggestive of BPH (LUTS/BPH) and treatment outcome have not been reported before. The aims of this study are to investigate the association between serum CRP levels and LUTS, and the role of serum CRP levels in predicting the medical treatment outcome of voiding or storage predominant LUTS in men.

## Materials and Methods

This study was a retrospective analysis and was approved by Buddhist Tzu Chi General Hospital Research Ethics Committee (TCGH IRB 099-03). Written informed consent was not obtained from patients because the study was retrospectively performed, which was specifically waived by the approving IRB. A total of 853 men visited the urologic clinic for LUTS/BPH were enrolled in this study. All patients should have LUTS, a TPV≥30 ml and completed the International Prostate Symptoms Score (IPSS) questionnaire and urological examinations. The parameters of uroflowmetry (maximum flow rate, Qmax), voided volume VV), post-void residual (PVR) volume, total prostate volume (TPV) and transition zone index (TZI) measured by transrectal ultrasound, serum prostate specific antigen (PSA) and serum CRP levels were obtained at the initial visit and after 3-month medical treatment. Serum CRP was measured using a Cobas Integra 400 AutoAnalyzer and a particle enhanced turbidimetric assay (Cobas Integra C-reactive protein Latex; Roche Diagnostics, Madison, WI). Assays were performed at the Hualien Tzu Chi General Hospital Central Laboratory. The lower limit of detection was 0.01 mg/dL and the normal range of the institute was 0.05 to 0.3 mg/dL. .Exclusion criteria included serum CRP level ≥3 mg/dL, PSA level ≥30 ng/ml without prostatic cancer, known urological malignancy, neurogenic bladder dysfunction and those taking non-steroid anti-inflammatory drugs or aspirin.

The patients were divided into three groups according to the severity of LUTS as mild (IPSS<8), moderate (8<IPSS<20) and severe (IPSS≥20) LUTS subgroups. The IPSS was further divided into voiding subscore (IPSS-V) and storage subscore (IPSS-S) and the voiding to storage subscore ratio (IPSS-V/S). Patients with IPSS-V/S>1 were considered as having emptying disorders and treated with doxazosin 4 mg QD. Patients with IPSS-V/S<1 were considered as having bladder storage disorders and treated with tolterodine 4 mg QD [13]. All patients underwent the medical treatment for 3 months. A successful treatment as defined as having a global response assessment (GRA) increase of 1 or more. Correlation analyses were performed between serum CRP levels with age, IPSS, TPV, TZI, Qmax, PVR, VV, PSA, between different LUTS subgroups, between baseline and post treatment, and between patients with a successful and failed treatment result.

Continuous variables are expressed as mean ± standard deviation, and categorical data are expressed as number and percentages. Statistical comparisons between the subgroups were tested using chi-square test for categorical variables, and the Wilcoxon rank sum test were used for continuous variables. Wilcoxon sign rank test was used to evaluate the significant difference of variables at baseline and after treatment. Pearson's correlation was implemented to measure the degree of association between two continuous variables. The variables that might affect the serum CRP levels were analyzed by univariate and multivariate logistic regression. All statistical assessments were 2-sided and considered significant at *p*<0.05. Statistical analyses were performed using SPSS version 15.0 statistical software (SPSS Inc., Chicago, IL, USA).

## Results

**Figure 1:**
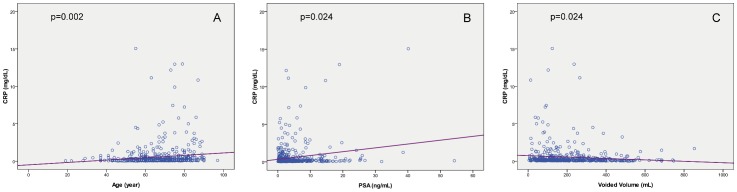
Serum CRP levels are significantly correlated with (A) age, (B) serum PSA levels, and (C) voided volume.

**Figure 2:**
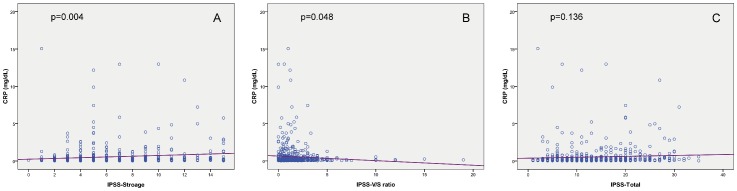
The serum CRP levels are significantly correlated with (A) storage IPSS and (B) IPSS V/S ratio, but not with (C) total IPSS.

The mean age was 66.9±11.6 years old and the mean serum CRP level was 0.31±0.43 mg/dL. In overall patients, serum CRP levels were significantly positively associated age (r^2^ = 0.139, p<0.001), the serum PSA levels (r^2^ = 0.098, p = 0.005), and negatively associated with VV (r^2^ = −0.087, p = 0.017) and Qmax (r^2^ = −0.08, p = 0.028) (**Fig. 1**). However, CRP levels were not significantly associated with TPV (r^2^ = −0.006, p = 0.854), TZI (r^2^ = −0.038, p = 0.278) or PVR (r^2^ = 0.068, p = 0.056). The mean IPSS was 13.6±7.2 in overall patients. When we correlated serum CRP levels with each item subscore of IPSS, a significant association was only noted in urgency (r^2^ = 0.133, p<0.001) and nocturia (r^2^ = 0.122, p<0.001), but not in the subscore of incomplete emptying (r^2^ = 0.015, p = 0.665), frequency (r^2^ = −0.003, p = 0937), intermittency (r^2^ = −0.003, p = 0.937), weal stream (r^2^ = 0.036, p = 0.297), straining to void (r^2^ = 0.025, p = 0.463) or quality of life index (r^2^ = 0.014, p = 0.678). Serum CRP levels were positively associated with total IPSS (r^2^ = 0.091, p = 0.008) and IPSS-S (r^2^ = 0.151, p<0.001), and negatively associated with IPSS-V/S ratio (r^2^ = −0.089, p = 0.014), but not with IPSS-V (r^2^ = 0.026, p = 0.457) (**Fig. 2**). The multivariate model was based on all significant variables in the univariate analysis and showed that only IPSS-S (p<0.001) and age (p = 0.004) were the independent predictors associated with an elevated serum CRP level. (**Table 1**)

**Table 1:** Multivariate analysis of variables significantly associated with an increased serum CRP level on univariate analysis.

Variable	Pearson correlation	Univariate p value	Multivariate p value
Age	0.139	<0.001	0.004
IPSS-Total	0.091	0.008	0.667
IPSS-V	0.026	0.457	
IPSS-S	0.151	<0.001	<0.001
IPSS-V/S	−0.089	0.014	0.398
TPV (ml)	−0.006	0.854	
TZI (%)	−0.038	0.278	
PSA (ng/ml)	0.098	0.005	0.063
Qmax (ml/s)	−0.80	0.028	0.308
Voided volume (ml)	−0.87	0.017	0.690
PVR (ml)	0.068	0.056	

IPSS: International Prostate Symptom Score. V: voiding subscore, S: storage subscore, V/S, voiding symptoms/storage symptoms, TPV: total prostate volume, TZI: transition zone index, PSA: prostate specific antigen, Qmax: maximum flow rate, PVR: postvoid residual.

Among the overall patients, 430 (50.4%) had voiding predominant LUTS (IPSS-V/S>1) and 423 (49.6%) had storage predominant LUTS (IPSS-V/S<1). The serum CRP levels at baseline were significantly higher in patients with IPSS-V/S<1 (0.35±0.50 mg/dL) than patients with IPSS-V/S>1 (0.27±0.34 mg/dL) (p = 0.012). Mild LUTS was noted in 219 patients (25.7%), moderate LUTS in 461 (54.0%), and severe LUTS in 173 (20.3%). The mean serum CRP level was significantly higher in severe LUTS group (0.40±0.52 mg/dL) than the moderate LUTS group (0.28±0.37 mg/dL) or mild LUTS group (0.31±0.44) (p = 0.008). Serum CRP level >0.30 mg/dL was noted in 220 (25.8%) and <0.30 mg/dL in 633 (74.2%). Compared with the patients of CRP<0.30 mg/dL, the patients with CRP>0.30 mg/dL had significantly greater age (66.2±11.1 v 69.1±12.6 years, p = 0.001), higher IPSS-V (6.78±5.28 v 7.90±5.91, p = 0.009), smaller voided volume (214±148 v 181±135 ml, p = 0.007) and smaller IPSS-V/S ratio (1.63±1.94 v 1.37±1.29, p = 0.027), however no significant difference was noted in TPV, TZI and PVR.

A total of 210 men who completed the 3 month medical treatment were available to follow up. After treatment with doxazosin for 3 months in 99 patients with IPSS-V/S>1, the IPSS parameters, uroflow parameters, and PSA levels all improved, but serum CRP levels did not change significantly. Among 111 patients with IPSS-V/S<1 and treated with tolterodine for 3 months significant improvements in total IPSS, IPSS-S and Qmax were noted. The serum CRP levels also showed significant decrease after treatment (0.36±0.53 v 0.21±0.35 mg/dL, p = 0.024) (**Table 2**).

**Table 2:** The changes of measured parameters at baseline and 3 months after medical treatment in patients with IPSS V/S>1 and V/S≤1.

	IPSS-V/S>1 Baseline (n = 99)	IPSS-V/S>1 3 months (n = 99)	IPSS-V/S≤1 Baseline (n = 111)	IPSS-V/S≤1 3 months (n = 111)
IPSS-total	16.8±6.81	8.46±5.82 *	9.47±5.92	7.04±5.04 *
IPSS-voiding	11.5±4.70	4.72±4.61 *	3.02±3.21	2.93±3.54
IPSS-storage	5.30±3.03	3.75±2.27 *	6.50±3.42	4.16±2.58 *
Qmax (ml/s)	10.2±5.23	12.1±5.75 *	11.6±7.15	13.4±8.46 *
Voided volume	212±120.	269±144 *	198±144	223±126
PVR (ml)	66.5±66.3	57.9±68.8	46.3±57.4	52.0±58.4.
TPV (ml)	47.8±21.2	47.1±21.0	56.2±36.3	53.9±32.6
TZI (%)	38.4±12.6	44.5±12.3	38.5±12.0	39.5±13.4
PSA (ng/ml)	3.57±4.66	2.52±2.75 *	5.35±5.27	4.79±5.10 *
CRP (mg/dl)	0.30±0.31	0.36±0.87	0.36±0.53	0.21±0.35 *

Data are presented as mean ± standard deviation,

indicates p<0.05 compared between baseline and 3 months after treatment.

IPSS: International Prostate Symptom Score. V: voiding subscore, S: storage subscore, V/S, voiding symptoms/storage symptoms, TPV: total prostate volume, TZI: transition zone index, PSA: prostate specific antigen, Qmax: maximum flow rate, PVR: postvoid residual.

## Discussion

Our study demonstrated that serum CRP levels in men with BPH and LUTS increased with age, higher PSA levels, the increased severity of LUTS, smaller voided volume, and higher storage symptoms. Multivariate analysis showed that IPSS-S and age were the independent predictors associated with an elevated serum CRP level, suggesting increased serum CRP level is associated with increased storage LUTS in men with BPH.

LUTS can result from a complex interplay of pathophysiologic features that include bladder dysfunction and bladder outlet dysfunction. The Male Attitudes Regarding Sexual Health (MARSH) study showed that the overall prevalence of LUTS was as high as 28% and storage symptoms are the most frequent subtype (13%) [14]. In the EPIC study, Irwin et al also found that storage symptoms were the most prevalent and bothersome symptoms [2]. In this study, 74.3% patients have moderate to severe LUTS. In addition, we found the storage symptoms are predominant as in 49.6% of men with LUTS and the severity of storage symptoms is associated with the higher serum CRP levels in patients. Based on these interesting findings, we suggest that serum CRP, which implies the inflammatory condition in subjects, is associated with storage symptoms rather than voiding symptoms in male LUTS.

IPSS-V/S ratio has been demonstrated to impact on phenotype of male LUTS and initial treatment options. Measuring IPSS subscores and calculating IPSS-V/S is a simple and useful method to differentiate failure to voiding and failure to storage lower urinary tract dysfunction (LUTD) in men with LUTS. When IPSS-V/S was used to differentiate male LUTS, failure to voiding LUTD was found in 81.2% of patients with IPSS-V/S>1, while failure to storage LUTD was found in 75.7% of patients with IPSS-V/S≤1 [15]. Using IPSS-V/S>1 in combination with TPV and Qmax provides a stronger predictor for bladder outlet-related LUTD than total IPSS score [16]. Initial treatment with doxazosin for patients with IPSS-V/S>1 and tolterodine for patients with IPSS-V/S≤1 has been shown safe and feasible. [13].

The results of this study demonstrate that serum CRP levels is significantly associated with urgency and nocturia subscore in IPSS, and can provide predictive value for differentiating IPSS-V/S>1 and IPSS-V/S<1 subgroups, however, serum CRP level cannot predict a successful treatment outcome for voiding predominant or storage predominant LUTS. It is possible that chronic inflammation might also exist in the prostate tissue in patients with IPSS-V/S>1. Medical treatment with alpha-blocker for 3 months could not reduce he serum CRP levels in patients with IPSS-V/S>1 although the clinical symptoms had improved. However, the inflammation in patients with IPSS-V/S<1 might exist in the bladder or other systems. Antimuscarinic therapy might provide a suppressive effect on both detrusor activity and suburothelial inflammation, therefore, both IPSS-S and serum CRP levels were significantly decreased. The same result had been demonstrated in the decrease of urinary nerve growth factor levels in OAB patients [17].

Our data differs from the findings of Boston Area Community Health (BACH) survey, which has emphasized the association between male nocturia and straining and CRP [18]. In the present study, the serum CRP levels positively associated with IPSS-S (p<0.001) and negatively associated with IPSS-V/S ratio (p = 0.014) but not the IPSS-V. Similarly, the results in the cohort study in Olmsted County also did not observe the association between of CRP level and the increase of obstructive LUTS [19]. According to the multivariate analyses in this study, serum CRP was only significantly associated with age and IPSS-S, suggesting that chronic inflammation increases with age and affects the bladder storage function that consequently causes decrease of voided volume and higher storage LUTS. However, the development of prostatic obstruction is not completely attributed to the chronic inflammation in the prostate tissue, the enlarged prostate glands are the most dominant portion to cause BOO and LUTS. Therefore, serum CRP levels are not associated with prostate and uroflow parameters in this cohort study. Although treatment with alpha-blocker can improve voiding LUTS, this medication might not alter the inflammatory condition in the prostate tissue and, therefore, cannot significantly reduce CRP levels after treatment.

Chronic inflammation has been considered as the aging process and has contribution to the development of prostate enlargement. Chronic inflammation is commonly seen in the prostate biopsy specimens, ranging from 43% to 98% [20], [21]. There is evidence of a relationship between the severity of LUTS and the degree of chronic inflammation in the prostate [8], [9]. In this study, although there is no biopsy specimen to validate the degree of chronic inflammation, the serum CRP level was found to increase in men with more severe LUTS. These findings suggest the degree of inflammation seems to associate with the severity of LUTS in the patients with moderate to severe LUTS/BPH. Among the patients with the mild and moderate LUTS, the elevation of serum CRP levels was also found. Patients with mild or moderate LUTS might attribute to bladder dysfunction rather than the outlet obstruction, therefore, a higher CRP level might indicate the presence of inflammation causing storage symptoms.

The role of serum CRP in bladder dysfunction has gained specific attention recently. Tyagi et al evaluated urinary cytokines in OAB patients and found that multiple cytokines related to inflammation were remarkably elevated in OAB patients than controls [22]. Their findings also supported the concept that chronic inflammation is one of the pathology of bladder storage failure. Our previous studies have shown serum CRP levels were significantly higher in subjects with OAB or interstitial cystitis/bladder pain syndrome than in controls. No significant difference in serum CRP level was noted between patients with OAB and IC/BPS. These results supported the association between chronic inflammation of the urinary bladder in patients with OAB or IC/BPS [23]. BACH survey also confirmed a consistent association of increasing CRP levels and OAB among both men and women [24]. In women with OAB-wet, higher serum CRP levels were also found and they were related to lower Qmax and higher body mass indices [25].

Male LUTS comprises bladder disorders and bladder outlet disorders. Patients with LUTS/BPH may have single or combined disorders. Since serum CRP indicates chronic inflammation, patients with elevated serum CRP levels and LUTS might have chronic inflammation in the lower urinary tract or other systems. Epidemiological studies have revealed a high prevalence rate of OAB in patients with metabolic syndrome, obesity, chronic heart failure and diabetes [26]–[28]. Systemic inflammation might also increase circulating cytokines and cause OAB [29], [30]. Therefore, medical treatment which can reduce inflammation might also improve OAB symptoms in male LUTS. Liao et al have found serum CRP levels were significantly associated with residual urgency in the patients with BPH after medical treatment. Patients with serum CRP levels ≥0.30 mg/dL had more urgency than those with serum CRP levels <0.30 mg/dL [31]. In the current study, the serum CRP levels were only decreased significantly in the patients with IPSS-V/S<1. This result further suggested that serum CRP levels are associated with storage LUTS, which might be due to some unknown mechanism of antimuscarinics on the inflammatory condition in bladder disorders.

## Conclusion

The serum CRP levels are associated with the storage predominant LUTS in the men with LUTS/BPH, suggesting the presence of chronic inflammation in men with LUTS/BPH. However, we failed to demonstrate the predictive role of serum CRP levels in differentiation between bladder disorders and bladder outlet disorders in male LUTS.
